# Multidisciplinary treatment for colorectal liver metastases in elderly patients

**DOI:** 10.1186/s12957-020-01950-4

**Published:** 2020-07-17

**Authors:** Taigo Hata, Yoshihiro Mise, Yoshihiro Ono, Takafumi Sato, Yosuke Inoue, Hiromichi Ito, Yu Takahashi, Katsuhiko Yanaga, Akio Saiura

**Affiliations:** 1 Department of Hepatobiliary Pancreatic Surgery, Cancer Institute Hospital, Japanese Foundation for Cancer Research, 3-8-31 Ariake, Koto-ku, Tokyo, 135-8550 Japan; 2 Department of Surgery, Jikei University School of Medicine, 3-19-18 Nishi-shinbashi, Minato-ku, Tokyo, 105-8471 Japan; 3 Department of Hepatobiliary-Pancreatic Surgery, Juntendo University School of Medicine, 3-1-3 Hongo, Bunkyo-ku, Tokyo, 113-8431 Japan

**Keywords:** Hepatectomy, Colorectal cancer, Elderly

## Abstract

**Background:**

Limited data describe the therapeutic practice and outcomes of colorectal liver metastases (CRLMs) in elderly patients. We aimed to evaluate the impact of age on multidisciplinary treatment for CRLMs.

**Methods:**

We reviewed treatment and outcomes for patients in different age groups who underwent initial hepatectomy for CRLMs from 2004 through 2012.

**Results:**

We studied 462 patients who were divided into three groups by age: ≤ 64 years (*n* = 265), 65–74 years (*n* = 151), and ≥ 75 years (*n* = 46). The rate of major hepatectomy and incidence of postoperative complications did not differ between groups. Adjuvant chemotherapy was used less in the ≥ 75-year group (19.6%) than that in the ≤ 64 (54.3%) or 65–74 age group (43.5%). Repeat hepatectomy for liver recurrence was performed less in the ≥ 75-year group (35%) than in the ≤ 64 (57%) or 65–74 (66%) age group. The 5-year disease-specific survival (DSS) rate of 44.2% in the ≥ 75-year group was lower than in the ≤ 64 (59.0%) or 65–74 (64.7%) age group. Multivariate analysis revealed age ≥ 75 years was an independent predictor of poor DSS.

**Conclusions:**

Liver resection for CRLMs can be performed safely in elderly patients. However, repeat resection for recurrence are performed less frequently in the elderly, which may lead to the poorer disease-specific prognosis.

## Introduction

With the rapid aging of the society, medical services for elderly patients have become more important in many developed countries. In Japan, the prevalence of colorectal cancer has continued to increase with the development of a super-aging society in which the population of those ≥ 65 years exceeds 21%, while the age-adjusted incidence rate of colorectal cancer has not changed [1–3]. Consequently, the treatment strategy for liver metastases, which are accompanied by more than 50% of colorectal cancer patients, has become a greater concern in elderly patients [4–17].

Hepatectomy is accepted as a cornerstone of treatment for colorectal liver metastases (CRLMs). Reports of encouraging long-term outcomes after surgery in the elderly with CRLMs have been published, with 5-year survival rates between 21 and 44% [4, 7, 14]. However, surgical treatment alone is not enough, and a multidisciplinary approach including repeat resection is now indispensable to achieve improved outcome for the treatment for CRLMs. Perioperative chemotherapy improves recurrence-free survival (RFS) in patients with CRLMs [18, 19], and aggressive repeat hepatectomy is also an important option because the first relapse after initial hepatectomy does not reflect cancer-related survival in patients with CRLMs [20–23]. So far, little is known about the relationship between advanced age and the prevalence of multidisciplinary treatment, such as perioperative chemotherapy or aggressive treatment for recurrence after initial hepatectomy.

The aim of this study was to investigate the impact of patient age on the feasibility of a multidisciplinary approach to CRLMs in a high-volume hepato-pancreato-biliary center in Japan. We examined the safety of initial hepatectomy, the prevalence of perioperative chemotherapy, recurrence pattern after initial hepatectomy, and the prevalence of repeat resection for the recurrence, in relation to the age.

## Material and methods

The prospectively maintained database of the Cancer Institute Hospital, Tokyo, Japan, was queried to identify patients who underwent initial hepatectomy for CRLMs at this hospital from January 2004 through December 2012. We excluded patients who underwent R2 resection. During the study period, 462 patients underwent initial hepatectomy for CRLMs with curative intent. Of these, 265 patients (57.4%) were ≤ 64 years of age with a median of 56 years; 151 patients (32.7%) were 65–74 years with a mean age of 68 years, and the other 46 patients (9.9%) were ≥ 75 years with a median age of 80 years. The study population was divided into three age groups: ≤ 64 years, 65–74 years, and ≥ 75 years of age. Baseline characteristics, perioperative course, and long-term outcomes were compared retrospectively between the three groups.

The Institutional Review Board of the Cancer Institute Hospital approved this study (Protocol 2018-1033).

### Indications for hepatectomy for CRLMs

In our institute, indications for resection of CRLMs during January 2004 through December 2012, period consisted of (1) no comorbid conditions that preclude hepatic resection, (2) all liver tumors that were amenable to resection would have a clear margin, leaving at least 30% of noncancerous remnant liver without a potentially ischemic or congested area, and (3) no unresectable extrahepatic tumors. The indications for repeat hepatectomy for liver recurrence were the same as those for initial hepatectomy. No age restriction was set for initial or repeat hepatectomy as long as patients met the above criteria.

Routine use of preoperative chemotherapy was not adopted until 2010. After 2010, preoperative chemotherapy was routinely performed for patients with ≥ 4 CRLMs or those with CRLMs > 50 mm or those with resectable extrahepatic metastases by imaging studies [24].

### Surgical procedure and postoperative outcomes

Parenchymal-sparing hepatectomy was the standard procedure regardless of the number or size of CRLMs. Major hepatectomy, which was defined as resection of ≥ 3 segments, was performed only when CRLMs were close to major Glisson’s pedicles. Following laparotomy and liver mobilization, fundamental intraoperative ultrasonography was performed to confirm the tumors detected by preoperative imaging and to search for new occult lesions. Resecting of all the tumors were intended, including newly detected nodules and disappearing CRLMs by preoperative imaging. Liver transections were performed by the crushing technique using the LigaSure vessel sealing system (Valleylab, Boulder, CO, USA), as reported previously [25]. Surgical margins were measured from the resected specimens. A positive surgical margin was defined as microscopic evidence of tumor at the resection margin.

The severity of postoperative complications was assessed according to the Clavien–Dindo classification; grade IIIa or worse was defined as a major complication. Any complications that developed within 90 days after the operation were included [26].

### Postoperative follow-up

Patient follow-up consisted of measuring serum tumor markers (carcinoembryonic antigen and carbohydrate antigen) at every visit as well as enhanced computed tomography every 3–6 months. Although adjuvant chemotherapy was not routinely administered, it was given to (1) patients who were included in clinical studies, (2) patients who underwent simultaneous resection of advanced primary disease, and (3) patients who had advanced CRLMs judged by a multidisciplinary team.

### Statistical analysis

Associations between variables with categorical data were sought using either Fisher’s exact test or Pearson’s chi-squared test. The Mann–Whitney’s *U* test was applied to continuous variables between the three groups. Survival curves were generated by the Kaplan–Meier method, and comparisons between the groups were performed using a log-rank test. Overall survival (OS), disease-specific survival (DSS), and recurrence-free survival (RFS) were defined as the interval from the date of primary hepatectomy to the date of all death, death attributed to colorectal cancer, and recurrence, respectively. Statistical significance was assessed using a two-tailed test across *p* < 0.05. All the statistical analyses were performed using the JMP software, version 10 (SAS Institute Inc., Cary, NC, USA).

## Results

### Patient characteristics

Baseline characteristics of the three groups are summarized in Table 1. The older age group of ≥ 75 years had more frequent hypertension and cardiovascular disease compared to the other groups.

**Table 1:** Characteristics of patients with CRLMs in the three age groups

Variable	≤ 64 years	65-74 years	≥ 75 years	*p* value
	(*n* = 265)	(*n* = 151)	(*n* = 46)	
*Patients*				
Age, year	56 (30-64)	68 (65-74)	80 (75-85)	
Sex, male, *n* (%)	162 (61.1)	104 (68.9)	33 (71.7)	0.163
*Primary tumor*				
Tumor differentiation, *n* (%)				
Well or moderately	244 (92.1)	134 (88.7)	41 (89.1)	0.991
Poorly	6 (4.0)	3 (2.0)	1 (2.2)	
Node, positive, *n* (%)	183 (69.6)	102 (68.5)	23 (51.1)	0.056
*Comorbidity*				
Hypertension, *n* (%)	46 (17.6)	37 (24.5)	19 (41.3)	0.001
Diabetes mellitus, *n* (%)	21 (7.9)	26 (17.2)	6 (13.0)	0.016
CNS conditions, *n* (%)	7 (2.6)	8 (2.3)	4 (8.7)	0.108
Pulmonary disease, *n* (%)	11 (4.2)	6 (4.0)	3 (6.5)	0.740
Cardiovascular disease, *n* (%)	9 (3.4)	11 (7.3)	8 (17.4)	0.0009
*Liver metastases*				
Synchronous with primary tumor, *n* (%)	151 (57.0)	82 (54.3)	23 (50)	0.642
Synchronous resection, *n* (%)	97 (36.6)	48 (31.8)	14 (30.4)	0.510
Serum CEA, ng/mL	6.6 (0.5-2606)	7.4 (0.9-7828)	9.1 (1.2-3097)	0.638
Size, cm	2.2 (0.3-19.0)	2.5 (0.2-10.5)	3.0 (1.1-10.0)	0.149
Number	2 (1-31)	2 (1-33)	2 (1-23)	0.041
Extrahepatic metastasis, *n* (%)	46 (19.3)	17 (12.5)	6 (14.3)	0.212

Continuous data expressed as median (range)

*CRLMs* colorectal cancer liver metastases; *CNS* central nervous system; *CEA* carcinoembryonic antigen

### Perioperative chemotherapy

Table 2 summarizes the perioperative course of the three age groups. No differences were found in the rate of administration of preoperative chemotherapy before hepatectomy. However, fewer patients ≥ 75 years of age (19.6%) received adjuvant chemotherapy after hepatectomy compared to the age groups of ≤ 64 years (54.3%) or 65–74 years (43.5%, *p* < 0.001).

**Table 2:** Perioperative course of patients with CRLMs in the three age groups

Variable	≤ 64 years	65-74 years	≥ 75 years	*p* value
	(*n* = 265)	(*n* = 151)	(*n* = 46)	
Major hepatectomy, *n* (%)	53 (20.0)	36 (23.8)	9 (19.6)	0.628
Operation time, min	305.0 (60-953)	275.0 (95-695)	263.0 (115-810)	0.194
Blood loss, ml	390 (20-2640)	300 (10-6530)	265 (5-1910)	0.023
Surgical margin: positive, *n* (%)	15 (5.7)	8 (5.3)	2 (4.3)	0.934
Preoperative chemotherapy, *n* (%)	107 (40.4)	47 (31.1)	12 (26.1)	0.057
Adjuvant chemotherapy, *n* (%)	144 (54.3)	65 (43.5)	9 (19.6)	< 0.001
*Complication*				
Major complications, *n* (%)	17 (6.4)	9 (6.0)	3 (6.5)	0.981
Biliary leakage, *n* (%)	9 (3.4)	6 (4.0)	2 (4.4)	0.926
Intraabdominal hemorrhage, *n* (%)	3 (1.1)	2 (1.3)	1 (2.1)	0.847
Surgical site infection, *n* (%)	24 (9.1)	14 (9.3)	3 (6.5)	0.837
Postoperative hospital stay, days	14 (5-160)	15 (8-372)	15 (8-45)	0.338
Mortality, *n*	0	0	0	

Continuous data expressed as median (range)

*CRLMs* colorectal cancer liver metastases.

### Surgical outcomes

The rate of major hepatectomy was not different between the three groups. Despite the same extent of surgery, the amount of blood loss was smallest in the ≥ 75 year group. No differences were found between the groups in the incidence of major complications.

### Recurrence pattern after initial hepatectomy

**Fig. 1:**
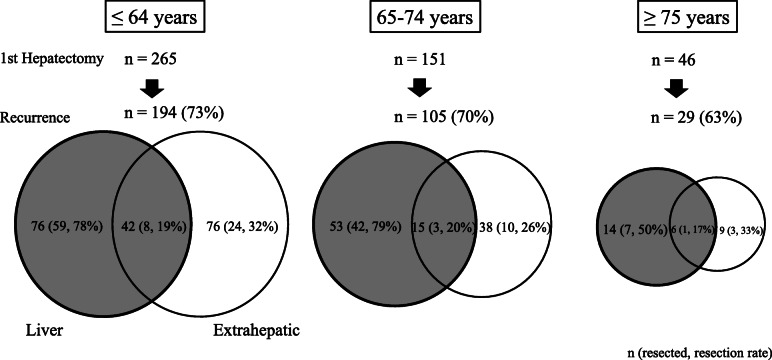
Recurrence patterns and resection rates for recurrence after initial hepatectomy in the three age groups. The numerals in the circles show the number and rate of patients who underwent repeat resection for recurrence

Figure 1 shows the recurrence sites in the 329 patients (71.2%) who developed recurrence after the initial hepatectomy. No differences were found in the incidence of liver recurrence between the three groups (*p* = 0.17). However, aged ≥ 75 years had less frequent repeat hepatectomy for liver recurrence (8/20 [35%]) as compared to the proportion in the age group of ≤ 64 years (67/118 [57%]) or 65–74 years (45/68 [66%]) (*p* = 0.0436). Among patients who had recurrence in ≥ 75 year group, DSS was better in those who had repeat resection for recurrence than that in those without repeat resection (*p* = 0.0024).

No 90-day mortality was found after repeat hepatectomy in any group.

### Long-term outcomes

**Fig. 2:**
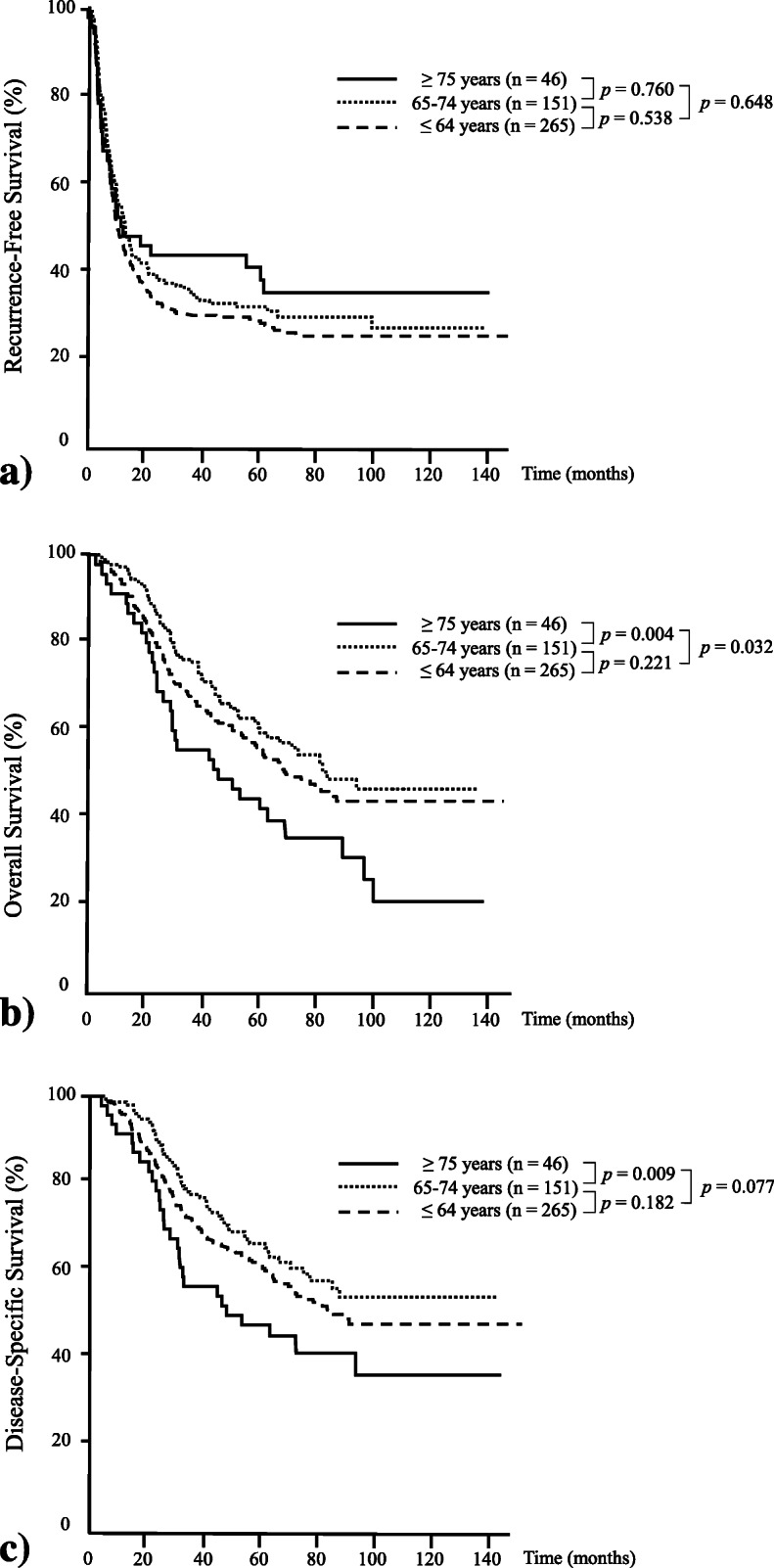
Survival curves in the three age groups. **a** Recurrence-free survival. **b** Overall survival. **c** Disease-specific survival

Figure 2a, b, and c demonstrates RFS, OS, and DSS curves of the three groups, respectively. The 3-year and 5-year RFS rate of the ≥ 75 year group (43.5%/37.9%) were lower than those in the ≤ 64 (29.8%/28.4%) or 65–74 groups (34.4%/31.7%, *p* = 0.4914). The 3-year and 5-year OS rates of the ≥ 75 year group (53.5%/44.2%) were lower than those in the ≤ 64 (69.5%/59.0%) or 65–74 groups (77.1%/64.7%, *p* = 0.0229). Furthermore, the 3-year and 5-year DSS rates of the ≥ 75 year group (53.5%/44.2%) were lower than those in the ≤ 64 (69.5%/59.0%) or 65–74 groups (77.1%/64.7%, *p* = 0.0187). The rates of mortality not related to the cancer in the < 65, 65-74, ≥ 75 groups were 4.2% (11/265), 4.6% (7/151), and 8.7% (4/46), respectively (*p* = 0.4080).

### Multivariate analysis of prognostic factors of DSS

Table 3 summarizes the results of univariate and multivariate analyses of clinical factors to predict impaired DSS in the study population. Age ≥ 75 years was found to be an independent prognostic factor of DSS (*p* = 0.0005, hazard ratio 3.23, confidence interval 1.72–5.75).

**Table 3:** Univariate and multivariate analysis of factors to predict impaired DSS in oldest patients with CLRMs

Variable		Univariate analysis	Multivariate analysis	
		*p* value	*p* value	HR (95%CI)
Patients	Sex, male	0.124		
	Age ≥ 75	0.038	0.0005	3.231 (1.725-5.758)
Preoperative factors	CEA ≥ 5	0.037		
	No preoperative chemotherapy	0.011		
Primary tumor	Site: rectum	0.245		
	N positive	< 0.01		
	Tumor differentiation: poorly	0.0061		
Liver metastases	Synchronous	0.932		
	Size ≥ 2 cm	0.0002		
	Number ≥ 2	< 0.01		
Perioperative factors	Non-PSH approach	0.049		
	Operative time > 180 min	0.004		
	Blood loss > 500	0.006		
	Surgical margin: positive	0.0003		
	Major complications	0.001		
Postoperative factors	DFI from 1st hepatectomy < 1 year	< 0.01	< 0.01	2.951 (1.778-5.163)
	No postoperative chemotherapy	0.94		

*CRLMs* colorectal cancer liver metastases; *DSS* disease-specific survival; *N* lymph node; *PSH* parenchymal-sparing hepatectomy; *DFI* disease-free interval

## Discussion

We assessed the relationship between advanced age and the outcome of multidisciplinary treatment for CRLMs. The study revealed that cancer-related survival in patients ≥ 75 years was significantly impaired, which may have been caused by the lower rate of repeat hepatectomy for liver recurrence in the older patients. However, liver resection can provide an acceptable prognosis with short-term outcomes comparable to those of the younger patients.

In the current study, the 5-year DSS rate in the elderly group (44.2%) was significantly lower than that of the younger patients. Multivariate analysis of the study population revealed that age ≥ 75 years was an independent predictor for impaired DSS. There are many reports assessing the prognostic benefit of hepatectomy in the elderly [5, 7, 9, 11, 14, 16, 17]. However, the mixed reporting of overall and cancer-specific survival has made it difficult to interpret the long-term outcome. To investigate the differences in chronological age, DSS needs to be assessed because OS is impaired in the elderly as a result of the more limited life expectancy than in younger patients. Brudvik et al. well demonstrated a significant difference between OS and DSS in the elderly (80–89 years of age) and the prognostic benefit of hepatectomy by showing that the gap between the 5-year survival of the age-matched national population (66.3%) and 5-year DSS rate (43.1%) decreases compared with the 5-year OS rate (32.5%) after hepatectomy [17].

Analysis of recurrence after initial hepatectomy revealed that repeat hepatectomy was performed less in the elderly group with liver recurrence, although the recurrence pattern was not different between the groups. This result indicated that in clinical practice, aggressive treatment for recurrence was reserved, even when indication criteria for repeat hepatectomy were met, regardless of the patient’s age. The major strength of this study was the detailed analysis of treatment for recurrence after initial hepatectomy, which revealed the cause of the poorer disease-specific prognosis in the elderly having CRLMs. Repeat hepatectomy is one of the essential treatments for CRLMs, because the biology of colorectal cancer is unique; the recurrence after the initial surgery is not directly associated with cancer-related death. Oba et al. demonstrated that the time to development of unresectable recurrence after initial surgery is a better surrogates prognosis than RFS in patients undergoing surgery for CRLMs [23]. The current study revealed that repeat hepatectomy was safely performed without mortality even in the elderly, which is consistent with a previous report showing no mortality in 114 patients ≥ 70 years having repeat resection after initial hepatectomy [7]. The results of the current study confirm that aggressive repeat resection is a feasible option to improve survival in well-selected older patients having recurrence after initial hepatectomy. The next step is to elucidate the indication process for repeat hepatectomy in the elderly so as to optimize patient selection for the aggressive treatment.

The prevalence of adjuvant chemotherapy after initial hepatectomy was lower in the elderly group than in the younger groups. This result may be another cause of poorer DSS in the elderly in this study. Adam et al. demonstrated that no adjuvant chemotherapy after initial hepatectomy was an independent predictor of reduced OS in the patients ≥ 70 years undergoing hepatectomy for CRLMs [7]. However, their analysis of OS rather than DSS has made it difficult to interpret the result because senile weakness intolerant to adjuvant chemotherapy may have been directly associated with cancer-unrelated death in their assessment. Although administration of adjuvant chemotherapy after hepatectomy was not selected as a prognostic factor of DSS in univariate and multivariate analysis in the current study, a recent randomized trial demonstrated that adjuvant chemotherapy improves RFS in patients undergoing hepatectomy for CRLMs [27]. Additional studies are needed to investigate the prognostic impact of adjuvant chemotherapy, especially in older patients for whom the balance between therapeutic effect and toxicity is of great importance.

Initial and repeat hepatectomies were safely performed in the elderly group without mortality in the current study. No differences were found in other short-term outcomes, such as the prevalence of major complications and the length of hospital stay after initial hepatectomy, even when surgical procedures were similar among the groups. We assume that the favorable short-term outcomes in the elderly were attributable to the low rate of major hepatectomy (19.6%) in this study, considering the results of previous reports. Although there is some discrepancy in the definition of “the elder” and “mortality,” previous large series of population-based or multicenter studies demonstrated the rates of major hepatectomy as 37.5% to 56% in the elderly. Consequently, the mortality rate was reportedly as high as 3.8% to 8% [7, 12, 14]. The parenchymal-sparing approach is now accepted as the standard procedure for resection of CRLMs to achieve better short- and long-term outcomes [28–30]. Greater concern should be taken to choose less invasive parenchymal-sparing hepatectomy in the elderly who are physically weak because of senile decay. Referral to a specialist hepatobiliary surgery team is favorable to avoid major hepatectomy because parenchymal-sparing hepatectomy for tumors in difficult locations is technically demanding [31].

The limitations of this study include its retrospective nature and the small number of patients in a single-center experience. As mentioned above, the selection process for repeat hepatectomy was not clear because of the limited data. In addition, the detailed data of administration of preoperative or adjuvant chemotherapy were not available in this study. However, the detailed analysis of the recurrence pattern and the treatment for recurrence would not have been possible using population-based data [12, 14]. In addition, although population-based analyses are said to better describe the outcomes achieved in routine practice [14], the trend in centralization of high-risk surgery is associated with improved short- as well as long-term outcomes [32–42]. Considering much better outcomes reported from high-volume liver centers [20, 43–45], the results demonstrated in the current study may reflect ideal practice in the near future when centralization is optimized for older patients with CRLMs undergoing hepatectomy.

In conclusion, in patients ≥ 75 years undergoing hepatectomy for CRLMs, cancer-related survival was significantly impaired, which may have been caused by the lower rate of repeat hepatectomy for recurrence in this population. However, liver resection can provide an acceptable prognosis with short-term outcomes comparable to those of the younger patients.

## Data Availability

The datasets used and/or analyzed during the current study are available from the corresponding author on reasonable request.
